# Tei Index Is the Best Echocardiographic Parameter for Assessing Right Ventricle Function in Patients With Unrepaired Congenital Heart Diseases With Outflow Tract Obstruction

**DOI:** 10.3389/fped.2018.00181

**Published:** 2018-06-26

**Authors:** Horacio Márquez-González, Mario H. Vargas, Lucelli Yáñez-Gutiérrez, Eduardo Almeida-Gutiérrez, Juan Garduño-Espinosa

**Affiliations:** ^1^Servicio de Cardiopatías Congénitas, Centro Médico Nacional Siglo XXI, Instituto Mexicano del Seguro Social, Mexico City, Mexico; ^2^Dirección de Investigación, Hospital Infantil de México Federico Gómez, Mexico City, Mexico; ^3^Unidad de Investigación Médica en Enfermdades Respirtorias, Hospital de Pediatría, Centro Médico Nacional Siglo XXI, Instituto Mexicano del Seguro Social, Mexico City, Mexico; ^4^Dirección de Educación e Investigación, Hospital de Cardiología, Centro Médico Nacional Siglo XXI, Instituto Mexicano del Seguro Social, Mexico City, Mexico

**Keywords:** congenital heart disease, childhood, right ventricle failure, cardiac catheterization, Tei index

## Abstract

**Objective:** Magnetic resonance imaging (MRI) and cardiac catheterization are diagnostic tools for right ventricle dysfunction (RVD), but those are expensive and often unavailable techniques. Thus, our objective was to identify clinical and/or echocardiographic variables capable of predicting a catheterization-based diagnosis of RVD.

**Design:** This was cross-sectional, diagnostic test accuracy study, considering the catheterization-based diagnosis of RVD as the gold standard.

**Patients:** Pediatric patients with non-repaired CHD with overload pressure were evaluated. Clinical variables (edema and functional class), transthoracic echocardiography (right heart dimensions, systolic and diastolic function, Doppler velocities), and cardiac catheterization (pressures and right ventricle systolic work measurements) were obtained during the same hospitalization.

**Results:** We included 253 patients with tetralogy of Fallot (39.9%), pulmonary atresia with ventricular septal defect (33.9%), type C Ebstein's anomaly (15.8%), or pulmonary stenosis (10.4%). Among clinical (vascular congestion, functional class derangement) and echocardiographic (indexed right ventricle diameter, fractional area change, tricuspid annular plane systolic excursion, S' wave, Tei index) variables, the Tei index (defined as the ratio of isovolumetric contraction time to ejection time) was the sole variable that exhibited high diagnostic capability, with 98.5% sensitivity, 97.4% specificity, 97.8% positive predictive value, and 98.3% negative predictive value, with 98.0% overall performance. Multivariate logistic regression confirmed that Tei index alone predicted the catheterization-based diagnosis of RVD.

**Conclusions:** Tei index is the best parameter that can be employed for the non-invasive identification of RVD in patients with CHD.

## Introduction

Currently, patients with congenital heart diseases (CHD) achieve more than 85% survival at 45 years of age, and this survival decreases according to the complexity of the cardiopathy (1). The majority of patients with CHD present heart failure, which constitutes the second cause of death (2), and right ventricle dysfunction (RVD) further increases morbidity and mortality (3).

Some CHD, such as tetralogy of Fallot, pulmonary atresia with ventricular septal defect (PA+VSD), pulmonary valve stenosis (PVS), and type C Ebstein's anomaly (4, 5) share the common obstructive factor of right ventricle outflow tract, which leads to right ventricle pressure overload. This mechanism, along with additional factors such volume overload secondary to left-to-right shunts, favors the development of RVD. Methods for identification of RVD vary among the published studies according to different populations and clinical settings. Thus, some authors have employed clinical variables (6, 7), while others have used laboratory biomarkers or echocardiographic variables (8).

Magnetic resonance imaging (MRI) is the best diagnostic tool for the evaluation of RVD, but it is expensive and not widely available. On the other hand, due to its capability for the evaluation of the morphology, functionality, and segmental pressures of the right ventricle, cardiac catheterization may be considered an equivalent of MRI (9). Because MRI is not always available and cardiac catheterization is an invasive method, transthoracic echocardiography (TTE) has been employed as an alternative method for the evaluation of RVD, mainly under conditions other than CHD (10–13). Thus, the usefulness of TTE for the evaluation of RVD in CHD merits full investigation. Our objective in the present study was to identify clinical and/or echocardiographic variables associated with a diagnosis of RVD based on cardiac catheterization.

## Methods

This was a prospective study carried out at the CHD Service of the Hospital de Cardiología, Centro Médico Nacional Siglo XXI, Instituto Mexicano del Seguro Social, in Mexico City during the 2010–2015 period. The protocol was reviewed and approved by our institutional scientific and bioethics committee (approval No. R-2014-3601-208). During hospitalization, prior to the catheterization procedure, the researcher responsible for the investigation provided the assent and consent letters, and the parents of the patients provided their written informed consent for their child's participation in this study.

The study population was composed of patients with a CHD and obstruction of the right ventricle output tract, such as tetralogy of Fallot, PA+VSD, PVS, and type C Ebstein's anomaly. They were inpatients of either sex, aged between 1 and 17 years, who were under preparation for total surgical repair. They were, during the same hospitalization, submitted to clinical evaluation and pre-surgical procedures including TTE and cardiac catheterization. These last procedures were performed by interventional cardiologists and echocardiographers certified in CHD and with ample experience in these procedures. Patients with non-programmed admission due to decompensation, with a prior non-palliative heart surgical procedure, with percutaneous valvuloplasty, or with coronary heart disease were excluded.

Cardiac catheterization was performed via a femoral vein or, alternatively, by subclavian or jugular vein. RVD was considered to be present when all of the following variables coincided: (1) right ventricle systolic pressure (RVSP, i.e., peak pressure recorded at the infundibulum during right ventricle contraction) >30 mmHg; (2) right ventricle diastolic pressure (RVDP, i.e., lowest pressure recorded into the right ventricle during relaxation) >12 mmHg, and (3) indexed right ventricle systolic work (RVSWi, i.e., the active work carried out during each contraction by the right ventricle, adjusted by body surface area) < 4 kg/m/m^2^. This latter parameter was calculated by the formula proposed by Chemla et al. (14), i.e., RVSWi = (MPAP − CVP) • RVSV • 0.0136, where CVP is the central venous pressure measured at the superior vena cava near its entry into the right atrium, and RVSV is the right ventricle systolic volume, also known as right ventricle stroke volume, calculated as the cardiac output measured by the thermodilution method divided by cardiac frequency. However, because mean pulmonary artery pressure (MPAP) may not correctly reflect the systolic work within the context of an outflow tract obstruction, we used the mean pressure of the right ventricle measured at the infundibulum level (i.e., *before* the outflow tract obstruction), instead of MPAP. In this calculation, we utilized the following formula: mean pressure of right ventricle at the infundibulum = (right ventricle systolic pressure at the infundibulum – right ventricle diastolic pressure at the infundibulum)/3 + right ventricle diastolic pressure at the infundibulum.

Clinical evaluation was conducted by pediatric cardiologists not participating in this research. The clinical variables analyzed comprised the derangement of Ross functional class by ≥1 class during the previous year, and congestive data including hepatomegaly (≥3 cm below the costal margin in children under age 2 years, or ≥2 cm below the costal margin in children aged between 2 and 5 years, or ≥1 cm below the costal margin in children aged >5 years of age) or jugular vein distention above two thirds of the neck. TTE was carried out by experienced pediatric cardiologists or echocardiography cardiologists and according to international guidelines (15). The following measures of right ventricle *systolic* function were obtained and evaluated: (1) Indexed right ventricle diameter, expressed in millimeters (average of triplicate measurement) and considered abnormal when *z*-value was greater than 2 of reference values adjusted by body surface area (16). (2) Right ventricle fractional area change (FAC), considered abnormal when lower than 35%. (3) Tei index, also known as right ventricular index of myocardial performance or myocardial performance index, was calculated with the use of pulsed Doppler as the ratio of isovolumetric contraction time to ejection time, and was considered abnormal when greater than 0.45. (4) Tricuspid annular plane systolic excursion (TAPSE), evaluated in M mode and referred as abnormal when the z score of the body surface-adjusted value was lower than −2 (17). (5) S' wave (pulsed Doppler peak velocity at the annulus), considered abnormal when the z score of the body surface-adjusted value was lower than −2 (18). Additionally, two measurements of right ventricle *diastolic* function were obtained: (1) The wave E′/A′ ratio, which was considered abnormal when <0.08. (2) Deceleration time, referred as abnormal if less than 23 m/s.

### Data analysis

We assessed the capability of clinical and echocardiographic variables to correctly diagnose RVD documented through cardiac catheterization. To this end, sensitivity, specificity, positive and negative predictive values, and overall performance were computed. Best cut-off point was evaluated through ROC curves. Multivariate logistic regression was employed to identify best predictor variables. Data was processed in Excel and SPSS v20 (IBM SPSS Statistics).

## Results

A total of 253 patients (57.7% males) with CHD and obstruction of the right ventricle output tract were studied. The main characteristics of this population can be observed in Table 1. Their age was 7.2 ± 0.17 years (mean ± standard error; range, 1.2–15 years), and tetralogy of Fallot (101 patients) was the most frequent cardiopathy, followed by PA+VSD (86 patients). According to evaluation by cardiac catheterization, 137 (54.2%) patients fulfilled the criteria of RVD and all underwent echocardiographic study (Supplementary Material). The CHD with highest frequency of RVD was type C Ebstein's anomaly (85% patients).

**Table 1:** Characteristics of 253 patients with congenital heart disease and obstruction of the right ventricle outflow included in the study.

**Characteristic**	**All patients**	**TOF**	**PA+VSD**	**EA**	**PS**
Number of patients	253	101	86	40	26
**GENDER**					
Male	146 (57.7)	58 (57.4)	48 (55.8)	28 (70)	12 (46.2)
Female	107 (42.3)	43 (42.6)	38 (44.2)	12 (30)	14 (53.8)
Age (years)	7.22 ± 0.17	7.18 ± 0.26	7.26 ± 0.30	7.86 ± 0.42	6.26 ± 0.38
Weight (kg)	25.30 ± 0.50	24.53 ± 0.70	25.16 ± 0.9	28.23 ± 1.39	24.27 ± 1.49
Height (cm)	117.02 ± 1.20	115.98 ± 1.78	116.60 ± 2.25	124.03 ± 3.16	111.69 ± 2.57
Body surface (m^2^/1.73)	0.90 ± 0.01	0.89 ± 0.02	0.90 ± 0.02	0.98 ± 0.04	0.86 ± 0.03
**CATHETERIZATION**					
RVSP (mmHg)	45.46 ± 1.44	42.41 ± 2.31	46.15 ± 2.57	53.55 ± 2.98	42.58 ± 4.24
RVDP (mmHg)	24.88 ± 1.46	19.59 ± 2.00	28.01 ± 2.76	33.00 ± 3.46	22.58 ± 4.73
RVSWi (kg/m/m^2^)	5.6 ± 0.57	4.3 ± 0.65	4.7 ± 0.67	4.3 ± 0.23	6.7 ± 0.34
Patients with RVD	137 (54.2)	44 (43.6)	45 (52.3)	34 (85)	14 (53.8)

*EA, Ebstein anomaly; PA+VSD, pulmonary atresia with ventricular septal defect; PS, pulmonary stenosis; RVD, right ventricle dysfunction; RVDP, right ventricle diastolic pressure; RVSP, right ventricle systolic pressure; RVSWi, indexed right ventricle systolic work; TOF, tetralogy of Fallot*.

When each potential predictor variable (congestive data, functional class, right ventricle diameter, right ventricle ejection fraction, S′, Tei index, and TAPSE) was evaluated for its capability to predict RVD, it was evident that the Tei index possessed, by far, the highest overall accuracy (>95%) in any of the four CHD studied (Table 2). This high predictive capability of the Tei index alone did not improve or even decrease when one or more additional variables were jointly evaluated. Because this latter analysis was performed with the Tei index dichotomized according to the cut-off value of 0.40, as proposed by international organizations (15), we explored whether this was indeed the best cut-off point to predict a catheterization-based RVD. Evaluation through the ROC curve yielded an area under the curve of 0.98 and the best cut-off point was indeed 0.40 (sensitivity, 98.5; specificity, 97.4).

**Table 2:** Accuracy of each individual variable for diagnosing RVD^*^ in patients with congenital heart disease.

	**Total**	RVD^*^	**Sensitivity**	**Specificity**	**PPV**	**NPV**	**Overall accuracy**
**EDEMA**							
TOF	57	24	54.5	42.1	42.1	54.5	47.5
PA+VSD	48	27	60.0	48.8	56.3	52.6	54.7
EA	15	15	44.1	100	100	24.0	52.5
PS	7	4	28.6	75.0	57.1	47.4	50.0
All patients	127	70	51.1	50.9	55.1	46.8	51.0
**ROSS FUNCTIONAL CLASS DERANGEMENT**							
TOF	32	6	13.6	54.4	18.8	44.9	36.6
PA+VSD	27	2	4.4	39.0	7.4	27.1	20.9
EA	3	3	8.8	100	100	16.2	22.5
PS	8	3	21.4	58.3	37.5	38.9	38.5
All patients	70	14	10.2	51.7	20.0	32.8	29.2
**RIGHT VENTRICLE DIAMETER**							
TOF	47	33	75.0	75.4	70.2	79.6	75.2
PA+VSD	44	31	68.9	68.3	70.5	66.7	68.6
EA	27	22	64.7	16.7	81.5	7.7	57.5
PS	19	13	92.9	50.0	68.4	85.7	73.1
All patients	137	99	72.3	67.2	72.3	67.2	70.0
**FRACTIONAL AREA CHANGE**							
TOF	48	29	65.9	66.7	60.4	71.7	66.3
PA+VSD	41	36	80.0	87.8	87.8	80.0	83.7
EA	26	26	76.5	100	100	42.9	80.0
PS	6	4	28.6	83.3	66.7	50.0	53.8
All patients	121	95	69.3	77.6	78.5	68.2	73.1
**S′ WAVE**							
TOF	43	26	59.1	70.2	60.5	69.0	65.3
PA+VSD	37	32	71.1	87.8	86.5	73.5	79.1
EA	22	22	64.7	100	100	33.3	70.0
PS	5	4	28.6	91.7	80.0	52.4	57.7
All patients	107	84	61.3	80.2	78.5	63.7	70.0
**Tei INDEX**							
TOF	45	44	100	98.2	97.8	100	99.0
PA+VSD	44	44	97.8	100	100	97.6	98.8
EA	34	33	97.1	83.3	97.1	83.3	95.0
PS	15	14	100	91.7	93.3	100	96.2
All patients	138	135	98.5	97.4	97.8	98.3	98.0
**TAPSE**							
TOF	43	31	70.5	78.9	72.1	77.6	75.2
PA+VSD	42	37	82.2	87.8	88.1	81.8	84.9
EA	22	22	64.7	100	100	33.3	70.0
PS	10	9	64.3	91.7	90.0	68.8	76.9
All patients	117	99	72.3	84.5	84.6	72.1	77.9
**DECELERATION TIME**							
TOF	14	3	6.8	80.7	21.4	52.9	48.9
PA+VSD	13	5	11.1	80.5	38.5	45.2	44.2
EA	6	5	14.7	83.3	83.3	14.7	25.0
PS	0	0	0	0	0	46.2	46.2
All patients	33	13	9.5	82.5	39.4	43.6	43.1
**E′/A′ RATIO**							
TOF	28	12	27.3	71.9	42.9	56.2	52.5
PA+VSD	12	8	17.8	90.2	66.7	50.0	52.3
EA	9	8	23.5	83.3	88.9	16.1	32.5
PS	8	5	35.7	75.0	62.5	50.0	53.8
All patients	57	33	24.1	79.3	57.9	46.9	49.4

* *RVD, right ventricle dysfunction as documented by cardiac catheterization. EA, Ebstein anomaly; NPV, negative predictive value; PA+VSD, pulmonary atresia with ventricular septal defect; PPV, positive predictive value; PS, pulmonary stenosis; TAPSE, tricuspid annular plane systolic excursion; Tei, Tei index or right ventricular index of myocardial performance; TOF, tetralogy of Fallot*.

When TTE parameters were entered into a multivariate logistic regression analysis as continuous variables, the final regression model retained Tei index as the sole variable capable of predicting the probability of having RVD (pRVD), with high accuracy, excluding from the model all other variables (congestive data, functional class, right ventricle diameter, FAC, TAPSE, and S′). The final model was pRVD = −27.563 + 71.766 • Tei index, with a pseudo *r*^2^ = 0.753.

## Discussion

The frequency of RVD in patients with non-repaired CHD is unknown, but it has been estimated that, during the immediate postoperative period and subsequent follow-up, right ventricle dysfunction can be found in up to 25–30% of patients (1). In the present work, cardiac catheterization diagnosed RVD in up to 54% of patients. A potential explanation for the high frequency of RVD observed in our study is that in Mexico, definitive repair of these congenital defects is usually carried out at more advanced ages (preschool or school age) than in developed countries (19), conditioning longer exposure to the phenomenon of pressure overload. Although cardiac catheterization is widely used for the initial diagnosis, it is not appropriate for patient follow-up.

Among the few studies that have assessed the value of TTE in CHD, Puchalski et al. (20) evaluated 22 patients with right-sided CHD and concluded that the reliability of echocardiography for identifying a moderately to severely diminished right ventricle systolic function was fair, with an adjusted kappa of 0.43. We found that abnormalities of right-ventricle morphology, specifically diastolic diameter, possessed overall diagnostic accuracy of 90% in cardiopathies such as type C Ebstein's anomaly and PA+VSD. This finding is consistent with the additional mechanism of diastolic overload occurring in these diseases, the former because of tricuspid valve insufficiency and the latter due to interventricular communication (21).

The Tei index exhibited highest diagnostic accuracy across all types of CHD studied. Song et al. (22) demonstrated that after repair of a CHD, the Tei index comprises a useful tool for the prediction of complications such as right ventricle dysfunction, and that this is the best non-invasive technique for postoperative evaluation of pulmonary valve insufficiency. Likewise, our results are agreement with the study by Bruch et al. (23) related to left-heart dysfunction. These authors performed cardiac catheterization and echocardiography in 32 patients with aortic stenosis, 10 of them with depressed systolic function. They found that a left Tei index greater than 0.42 had very good diagnostic accuracy, with 100% sensitivity and 91% sensitivity to predict a depressed systolic function.

With respect to the remaining echocardiographic variables, such as tricuspid annular motion and TAPSE, these proved to possess an overall accuracy lower than 80%. This might be explained because these parameters are more reliable for evaluation of CHD with pulmonary hyperflow, as demonstrated by Koestenberger et al. (17) in pediatric patients with tetralogy of Fallot and pulmonary hypertension. These authors found that, during RVEF assessment, correlation between TAPSE and MRI was better in patients with CHD and volume overload (*r* = 0.81) as compared with patients with tetralogy of Fallot (*r* = 0.65) (17). On the other hand, in 2016, Anavekar et al. (24) found that right ventricle FAC measured by TTE had good correlation (*r* = 0.80) with MRI-derived right ventricle ejection fraction in 36 adult patients submitted for evaluation of ischemia or viability. The authors suggested that this method could be utilized for the assessment of right ventricle function in the clinical setting, a suggestion that has been supported by others (25). In our study, we found relatively good overall performance of FAC (73.1%). However, even this proportion was much lower than that achieved by the Tei index (98.0%); therefore, this latter parameter should be preferred over FAC for assessment of RVD in patients with CHD.

The two echocardiographic measurements employed for evaluating right ventricle diastolic function exhibited lower diagnostic accuracy, probably because the major component of cardiopathies included in the study was obstructive in nature.

In the present study, the two clinical variables analyzed (vascular congestion and Ross functional class derangement) demonstrated low diagnostic accuracy in the majority of CHD. However, both clinical variables yielded a specificity of 100% among patients with type C Ebstein's anomaly. This may be explained because criteria for cardiac catheterization in Ebstein's anomaly comprises functional class derangement and low oxygen saturation (caused by a right-to-left shunt due to high pressures inside the right atrium) (26). Borgdorff et al. (27) demonstrated, in rat models with pulmonary artery banding, that long-term exposure to pressure overload produces vascular congestion and difficult breathing and induces significant changes in the systolic and diastolic function of the right ventricle.

The major limitation of our study was that the comparator for TTE was cardiac catheterization and not MRI, which at present is considered the gold standard for RVD evaluation. Likewise, we did not perform more sophisticated tests, such as strain rate or torsion, and in our RVD definition, we included RVSWi as indicator of contractile deficiency but, though reasonable, applicability of this parameter to CHD has not yet been corroborated. Conversely, advantages of our study included that the methodology employed warranted that both procedures (cardiac catheterization and TTE) were performed with a short lag time (during the same hospitalization), and that patients were devoid of any ventricular damage secondary to surgical procedures, guaranteeing that the evaluation focused only on hemodynamic status conditioned by the CHD.

## Conclusions

In conclusion, the Tei index is a useful echocardiographic parameter for diagnosing RVD in pediatric population with CHD. This index is non-invasive, relatively easy to obtain, and can be repeated as many times as needed during the patient's follow-up. In countries where RMI and cardiac catheterization are not easily available, the Tei index may help clinicians to perform timely identification of RVD in order to modify treatment and achieve a better prognosis.

## Author contributions

All authors contributed substantially to the conception and design of the study, and to the critical review of the manuscript. LY-G performed the majority of echocardiographic evaluations. HM-G was the main individual responsible for the project and was involved in the full data collection and, along with MV, in data analysis and in drafting of the manuscript.

### Conflict of interest statement

The authors declare that the research was conducted in the absence of any commercial or financial relationships that could be construed as a potential conflict of interest. The reviewer GJS and handling Editor declared their shared affiliation.
